# A novel functional gene associated with cold tolerance at the seedling stage in rice

**DOI:** 10.1111/pbi.12704

**Published:** 2017-03-30

**Authors:** Junliang Zhao, Shaohong Zhang, Jingfang Dong, Tifeng Yang, Xingxue Mao, Qing Liu, Xiaofei Wang, Bin Liu

**Affiliations:** ^1^ Rice Research Institute Guangdong Academy of Agricultural Sciences Guangzhou China; ^2^ Guangdong Provincial Key Laboratory of New Technology in Rice Breeding Guangdong Academy of Agricultural Sciences Guangzhou China

**Keywords:** rice, quantitative trait locus (QTL), cold tolerance, QTL mapping, expression profiling, gene cloning

## Abstract

Identification and cloning of cold‐tolerant genes that can stably express under different cold environments are crucial for molecular rice breeding for cold tolerance. In the previous study, we identified a cold‐tolerant QTL at the seedling stage, *qCTS‐9* which could be detected under different cold environments using a recombinant inbred line (RIL) population derived from a cold‐tolerant variety Lijiangxintuanheigu (LTH) and a cold‐sensitive variety Shanhuangzhan 2 (SHZ‐2). In this study, eight candidate genes within the *qCTS‐9* interval were identified through integrated analysis of QTL mapping with genomewide differential expression profiling of LTH. The qRT‐PCR assay showed that only Os09g0410300 exhibited different expression patterns between LTH and SHZ‐2 during cold stress, and significantly positive correlation was found between cold induction of Os09g0410300 and seedling cold tolerance in the RI lines. Five SNPs and one InDel in the promoters of Os09g0410300 were detected between LTH and SHZ‐2, and the InDel marker ID410300 designed based on the insertion–deletion polymorphism in the promoter was significantly associated with seedling cold tolerance in RIL population. Further, Os09g0410300 over‐expression plants exhibited enhanced cold tolerance at the seedling stage compared with the wild‐type plants. Thus, our results suggest that Os09g0410300 is the functional gene underlying *qCTS‐9*. To our knowledge, it is a novel gene contributed to enhance cold tolerance at the seedling stage in rice. Identification of the functional gene underlying *qCTS‐9* and development of the gene‐specific marker will facilitate molecular breeding for cold tolerance at the seedling stage in rice through transgenic approach and marker‐assisted selection (MAS).

## Introduction

Due to its origin in tropical and subtropical regions, rice is more sensitive to cold stress than other cereal crops. Low temperatures comprise a major climatic problem for rice growing in 25 countries (Cruz *et al*., 2013). According to the statistic, low temperature affects more than 15 million hm^2^ of rice worldwide (Sthapit and Witcombe, 1998). Cold damage can occur at different developmental growth stages in rice. Chilling injury at the seedling stage can lead to leaf discoloration or yellowing, leaf rolling or wilting, slowed growth, delayed crop maturation, poor establishment and, subsequently, decrease in yield. Therefore, cold tolerance at the seedling stage has been one of the main targets in rice breeding.

Development and use of cold‐tolerant variety has been considered as the most economical and effective way to avoid low temperature damage in rice. However, rice breeding for cold tolerance is difficult due to its polygenic nature and inadequate knowledge on the genetic basis of cold tolerance. Since the development of molecular marker technology in the early 1990s, much effort has been made in identification of genes associated with cold tolerance in rice. So far, more than 80 quantitative trait loci (QTLs) for cold tolerance at the seedling stage in rice have been identified (Andaya and Mackill, 2003; Han *et al*., 2004, 2007; Koseki *et al*., 2010; Lou *et al*., 2007; Suh *et al*., 2012; Zhan *et al*., 2005; Zhang *et al*., 2014). However, few successful cases have been reported in marker‐assisted selection (MAS) for cold tolerance at the seedling stage in rice. The unstable expression for most of the cold‐tolerant QTLs under different low temperature conditions could be one of the reasons related to this issue. Andaya and Mackill (2003) identified five and six QTLs for cold tolerance at the seedling stage under constant 9 °C and 25/9 °C (day/night) growth in the growth chamber, respectively, but only one QTL was detected in both cold environments. Our previous study (Zhang *et al*., 2014) also showed that four QTLs for cold tolerance at the seedling stage were detected on chromosome 1, 6, 9 and 12 under cold water irrigation, while five QTLs were detected on chromosomes 7, 8, 9, 11 and 12 when subjected to cold stress in the low temperature growth chamber. However, only two QTLs, *qCTS‐9* and *qCTS‐12,* were detected in both cold conditions. These results suggest that although a few QTLs for cold tolerance at the seedling stage are relatively stable in different cold environments, most of them are environment‐specific. Thus, use of unstable QTLs will frustrate MAS for cold tolerance in practical application. In addition, inaccurate mapping of QTL is another main factor limiting the application of MAS for cold tolerance at the seedling stage. A prerequisite for successful MAS for QTL is the knowledge of the predictive value of the markers. However, most of the QTLs including the cold‐tolerant QTLs in rice have been mapped to a region of 10–30 cM using primary populations (Salvi and Tuberosa, 2005) and recombination between markers and the target gene is expected to occur when MAS is performed (Collard *et al*., 2005). This issue can be only addressed by fine mapping, particularly, isolation of the genes underlying the QTLs. Therefore, identification, fine mapping and cloning of the QTLs that can stably express in different cold environments are the key for efficient MAS or transgenic breeding for cold tolerance at the seedling stage.

Positional cloning strategy has been widely used for isolation of major genes in plants. It has been also successfully used for isolating the functional genes of some major effect QTLs, such as the QTLs controlling heading date, salt tolerance, grain shape and grain weight in rice (Fan *et al*., 2006; Ren *et al*., 2005; Yano *et al*., 2000). However, although it is straight forward and effective approach for gene cloning, typical positional cloning processes are labour‐intensive, time‐consuming and costly. After obtained the target QTL from primary mapping, another large population with several thousand lines must be developed to create enough recombinants in the target region for further fine mapping. Then, phenotypic evaluation and genotyping of these lines are conducted. It takes about 3–5 years to complete all these processes. That is why only a few genes underlying QTLs have been cloned, although numerous QTLs are identified in plants. Furthermore, positional cloning has been limited exclusively to major QTLs which explain the phenotypic variations higher than 15% in the primary mapping (Salvi and Tuberosa, 2005) and this approach may not be the best choice to isolate the functional genes from minor effect QTLs (Hu *et al*., 2008). With the rapid development of genomic technology, microarray emerges as a powerful genomic tool. It has been extensively applied to dissect the quantitative traits such as cold tolerance at the seedling stage in rice (Rabbani *et al*., 2003; Zhang *et al*., 2012; Zhao *et al*., 2015). These results prove that microarray‐based expression profiling is a high‐throughput approach to identify functional genes and dissect the pathways related to the target traits. However, expression profiling generally results in a large number of differentially expressed genes (DEGs) due to the crosstalk and overlapping pathways of genes involved in quantitative traits. It is difficult to tell which genes are important for the target trait. Wayne and McIntyre (2002) proposed to integrate QTL mapping with arraying to make good use of the merits of the two approaches for identification of functional genes of QTLs. This integrated approach combines the target orientation and accuracy of positional cloning and the high throughput of genomewide transcription analysis, leading to a significant reduction of the number of candidate genes. This approach has been proved to be an efficient method in identification of functional genes underlying QTLs in plants (Deshmukh *et al*., 2010; Liu *et al*., 2013; Yano *et al*., 2012).

Our previous study (Zhang *et al*., 2014) identified seven QTLs for cold tolerance at the seedling stage using a recombinant inbred line (RIL) population derived from a cross between the cold‐tolerant variety Lijiangxintuanheigu (LTH) and the cold‐sensitive variety Shanhuangzhan 2 (SHZ‐2) under 9 °C cold water irrigation and growth in 11 °C low temperature growth chamber. Among the seven QTLs, *qCTS‐9* could be detected using different evaluation indicators under two distinct cold environments. Particularly, it is noteworthy that the *qCTS‐9* is overlapped with the previously identified QTL detected at both 4 °C and 12 °C using different germplasm (Liu *et al*., 2010). It seems that *qCTS‐9* may express in different genetic backgrounds and different cold environments. This QTL should have a great potential value in rice breeding for cold tolerance at the seedling stage. In this study, an integrated approach of combining QTL mapping and genomewide expression profiling was applied to identify the functional genes underlying *qCTS‐9*. We are able to quickly identify Os09g0410300 as the candidate gene of *qCTS‐9*. To validate the function of Os09g0410300 on cold tolerance at the seedling stage, the correlation between cold induction of Os09g0410300 and seedling cold tolerance in RI lines derived from LTH and SHZ‐2 was analysed. Further functional confirmation of Os09g0410300 was conducted using over‐expression transgenic method. Through these analyses, we were able to identify and confirm that Os09g0410300 is the functional genes underlying *qCTS‐9*. To our knowledge, Os09g0410300 is a novel functional gene responsible for cold tolerance at the seedling stage in rice. Our results suggest that integrating QTL mapping with genomewide differential expression profiling is an efficient approach to identify the genes associated with complex traits such as cold tolerance in rice, and identification of the functional gene underlying *qCTS‐9* in this study should have a potential application in both MAS and transgenic breeding for cold tolerance at the seedling stage in rice.

## Results

### Delimitation of confidence interval of the cold‐tolerant QTL *qCTS‐9*

**Figure 1:**
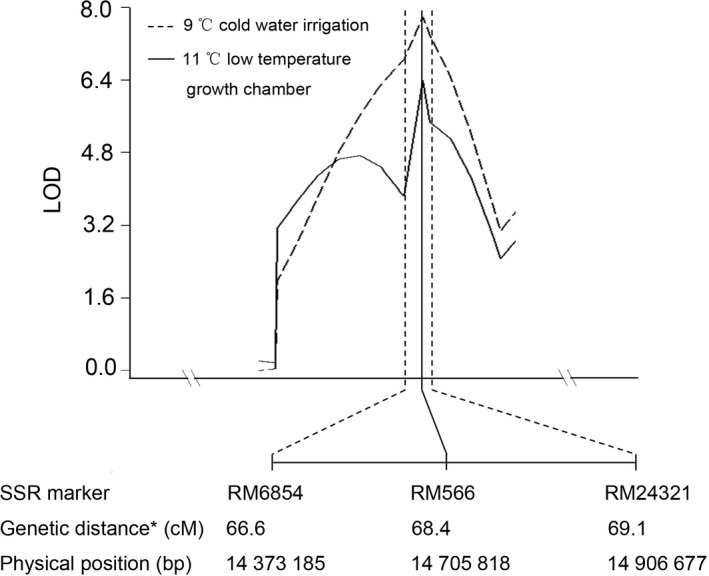
LOD score plots showing location of *qCTS‐9* on chromosome 9 identified under 9 °C cold water irrigation and growth in 11 °C low temperature growth chamber. The phenotypic data of cold tolerance are from our previous study (Zhang *et al*., 2014). The peak of each LOD curve indicates where *qCTS‐9* is most likely to be located. *The genetic distance was based on the RIL population derived from LTH and SHZ‐2.

In our previous study (Zhang *et al*., 2014), *qCTS‐9* was mapped to a region spanned over RM6854‐RM566‐RM434 on chromosome 9. RM566 was the peak position of *qCTS‐9*. The marker RM6854 was the left boundary of the confidence interval of *qCTS‐9*, while the right boundary was located between RM566 and RM434. To delimitate the right boundary of the confidence interval, more polymorphic SSR markers located between RM566 and RM434 were used for genotyping of RIL population derived from the LTH and SHZ‐2. Finally, *qCTS‐9* was defined to a 534 kb confidence interval covering three SSR markers, RM6854‐RM566‐RM24321 on chromosome 9. RM566 was still the most significant marker and the peak position of *qCTS‐9* (Figure 1). Within the defined QTL region, 58 genes were predicted based on RAP‐DB database (http://rapdb.dna.affrc.go.jp/) (Sakai *et al*., 2013) (Table S1).

### Differentially expressed genes identified within the interval of *qCTS‐9*

To cut down the number of candidate genes inferred from QTL mapping, we performed microarray‐based genomewide gene expression profiling of the cold‐tolerant variety LTH at 6, 12, 24 and 48 h after cold stress in our previous study. A total of 5557 DEGs were identified at least at one time point after cold stress using the combined criteria of threefold change and a cut‐off of *q*‐values (≤0.05) (Zhao *et al*., 2015). Among the 58 predicted genes within the defined interval of *qCTS‐9*, 6 genes were differentially expressed at least at one time point during cold stress (Tables 1 and 2). In order not to loss possible candidate genes, two genes (Os09g0412200 and Os09g0413000) within *qCTS‐9* interval showing twofold change during cold stress were also included. Thus, eight DEGs within the interval of *qCTS‐9* were selected as candidate genes for further confirmation (Table 1).

**Table 1:** Candidate genes in *qCTS‐9* interval

RAP Locus	RAP Annotation
Os09g0410300	Conserved hypothetical protein
Os09g0412200^a^	Protein of unknown function DUF246 plant family protein
Os09g0412400	Conserved hypothetical protein
Os09g0412700	Conserved hypothetical protein
Os09g0413000^a^	Hypothetical protein
Os09g0413700	Conserved hypothetical protein
Os09g0415700	Methyltransferase putative family protein
Os09g0416200	Similar to glucose transporter (Fragment)

Candidate genes were identified based on differential expression of the predicted genes in the interval of *qCTS‐9* in LTH after cold treatment using the combined criteria of threefold change and a cut‐off of *q*‐values (≤0.05).

a Genes showed twofold change during cold stress. RAP Locus and RAP annotation are based on the Rice Annotation Project Database (RAP‐DB): http://rapdb.dna.affrc.go.jp/.

**Table 2:** Expression changes of the eight candidate genes in LTH and SHZ‐2 after cold stress measured by microarray and real‐time PCR

Candidate genes	6 h			12 h			24 h			48 h		
	Microarray LTH	qRT‐PCR LTH	qRT‐PCR SHZ‐2	Microarray LTH	qRT‐PCR LTH	qRT‐PCR SHZ‐2	Microarray LTH	qRT‐PCR LTH	qRT‐PCR SHZ‐2	Microarray LTH	qRT‐PCR LTH	qRT‐PCR SHZ‐2
Os09g0410300	2.49^a^	3.85	1.67	4.29	7.18	1.51	2.25	2.85	0.72	1.49	0.94	0.48
Os09g0412200	0.94	1.93	1.42	0.64	1.64	0.85	0.40	0.40	0.61	0.44	0.56	0.24
Os09g0412400	0.35	0.74	1.08	0.48	0.68	0.92	0.37	0.45	0.3	0.13	0.21	0.11
Os09g0412700	0.79	1.52	0.31	0.46	1.28	1.05	0.11	0.16	0.14	0.17	0.08	0.05
Os09g0413000	1.38	2.61	1.78	2.01	2.52	2.30	1.30	1.63	1.12	1.48	0.85	0.52
Os09g0413700	0.79	1.02	1.09	0.93	1.48	1.59	0.26	0.40	0.40	0.17	0.11	0.09
Os09g0415700	1.74	1.03	0.59	1.68	1.71	1.81	5.56	2.98	1.91	2.61	2.05	1.75
Os09g0416200	2.92	2.38	0.92	1.61	3.77	5.63	2.40	5.35	2.44	3.73	3.99	2.22

a Fold change: expression data of cold treatment/expression data of control.

### Differential expression analysis of candidate genes in two parents and their derived RI lines

To confirm the results from microarray and select the most possible candidate gene for further functional confirmation, expression patterns of the eight candidate genes in the two parents under cold stress were analysed using qRT‐PCR. Similar expression patterns of the eight candidate genes in LTH were detected by both microarray and qRT‐PCR (Table 2), suggesting the reliability of our microarray experiments. By comparing the expression patterns of the eight candidate genes in the cold‐tolerant parent LTH and the cold‐sensitive parent SHZ‐2, it was found that all genes except for Os09g0410300 exhibited similar expression pattern between LTH and SHZ‐2 during cold stress. The expression level of Os09g0410300 increase three times at 6 h, even seven times at 12 h after cold treatment compared with control in LTH. In contrast, no significant change in expression (lower than twofold) of this gene was observed in SHZ‐2 during cold stress (Table 2). Therefore, Os09g0410300 was considered as the most possible functional gene underlying *qCTS‐9*.

**Figure 2:**
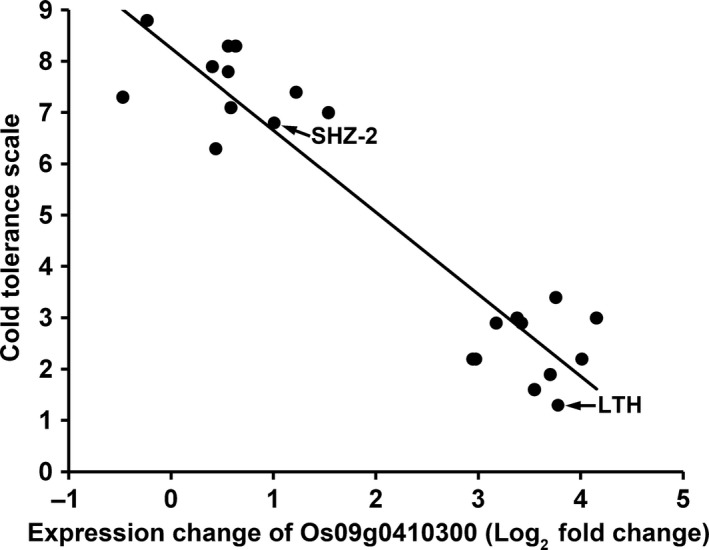
Correlation between the expression change of Os09g0410300 and cold tolerance in RI lines after cold treatment. The expression change of Os09g0410300 (fold change: expression data of cold treatment/expression data of control) at 12 h after cold treatment was log (base 2)‐transformed. Cold tolerance after cold treatment for 5 days was measured using the scale of 1 (tolerant, all leaves normal, no apparent visual injury) to 9 (sensitive, all leaves rolled and wilted, seedlings apparently dead). Two parents (LTH and SHZ‐2) were included.

To further validate the function of Os09g0410300 on cold tolerance, we analysed the relationship between the cold inducibility of Os09g0410300 and the seedling cold tolerance in RI lines derived from LTH and SHZ‐2. Twenty RI lines consisting of 10 cold tolerance lines and 10 cold‐sensitive lines based on the evaluation of cold tolerance at the seedling stage in our previous study (Zhang *et al*., 2014) were selected for Os09g0410300 expression assay. The cold‐tolerant phenotypes of the RI lines and the expression level of Os09g0410300 after cold treatment are shown in Table S2. At 12 h after 11 °C cold treatment, the expression of Os09g0410300 had twofold induction in SHZ‐2, while 13‐fold induction was observed in LTH. Os09g0410300 in the 10 cold tolerance lines was significantly up regulated with expression changes ranging from sevenfold to 17‐fold. However, no significant up‐regulation was observed in the 10 cold‐sensitive lines with a cut‐off of threefold change. Highly significant correlation was found between cold induction of Os09g0410300 and seedling cold tolerance in the RI lines (*r* = 0.8807, *P* < 0.0001; Figure 2).

### Cloning and sequence analysis of Os09g0410300 from LTH and SHZ‐2

**Figure 3:**
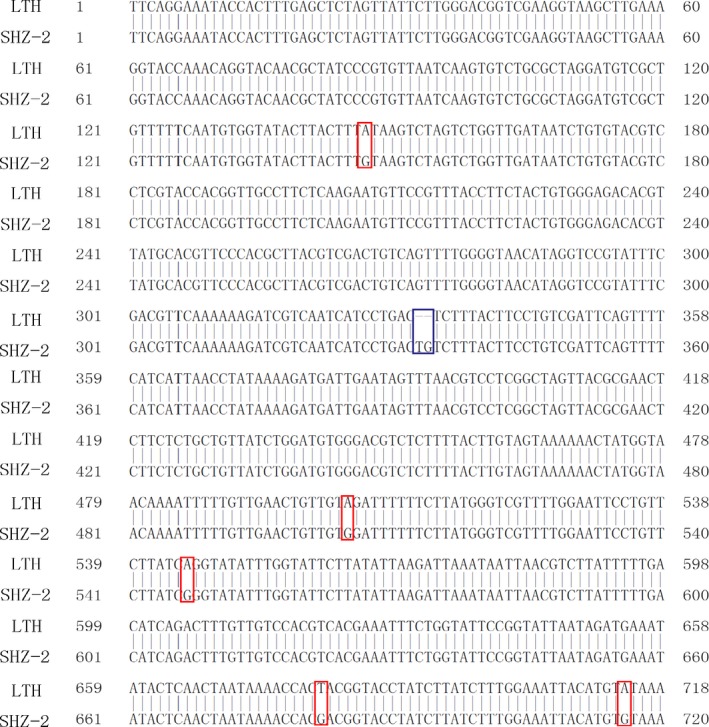
The five SNPs and one InDel in the promoters (1500 bp upstream) of Os09g0410300 between LTH and SHZ‐2. Sequences are indicated as reverse format. The red and blue boxes indicate the SNPs and InDel between LTH and SHZ‐2, respectively.

To find additional evidence to support the possible function of Os09g0410300, the gene from LTH and SHZ‐2 was cloned and sequenced. Sequence analysis revealed that no difference was detected in the cDNA sequence of Os09g0410300 between LTH and SHZ‐2. However, in the promoter region (1.5 kb upstream of the predicted transcription initiation site), five SNPs and one InDel were detected between LTH and SHZ‐2 (Figure 3). *Cis*‐element analysis in the Os09g0410300 promoters by PLACE (http://www.dna.affrc.go.jp/PLACE/) reveals 7 *cis*‐elements differences between LTH and SHZ‐2 (four specific for LTH and three specific for SHZ‐2) (Table S3). Based on their annotations, they are not the confirmed cold‐inducible elements.

### Association between the difference in Os09g0410300 promoter sequence and the cold tolerance at the seedling stage in RIL population

To further validate the association between the difference in Os09g0410300 promoter sequence and cold tolerance at the seedling stage, an InDel marker ID410300 was designed based on the 2 bp insertion–deletion polymorphism in the promoter of Os09g0410300 between LTH and SHZ‐2 (Figure 3 and Table S4), and genotyping was conducted in the RIL population consisting of 204 lines. Single‐factor analysis of variance (GLM procedure in SAS software; SAS Institute, 2000) revealed that ID410300 was significantly associated with cold tolerance at the seedling stage in RIL population under cold stress (*P* < 0.0001, Table S5).

### Functional confirmation of Os09g0410300 by over‐expression transformation

**Figure 4:**
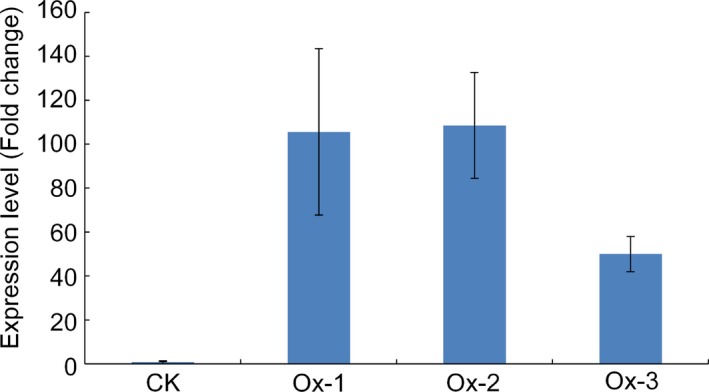
Expression levels of Os09g0410300 in the three independent over‐expression transgenic lines. Fold change: expression data of over‐expression transgenic line/expression data of CK. CK represents nontransgenic wild‐type plants; Ox‐1, Ox‐2 and Ox‐3 represent the three independent over‐expression transgenic lines, respectively.

To further confirm the function of Os09g0410300 in cold tolerance at the seedling stage in rice, full‐length cDNA of Os09g0410300 was cloned from LTH and over‐expressed in Zhonghua‐11 (ZH‐11) under the control of a CaMV 35S promoter. Three homozygous T2 transgenic lines (Ox‐1, Ox2 and Ox‐3) were selected for characterization. The qRT‐PCR assay showed that all three transgenic lines displayed much higher expression levels of Os09g0410300 than nontransgenic wild type (Figure 4).

**Figure 5:**
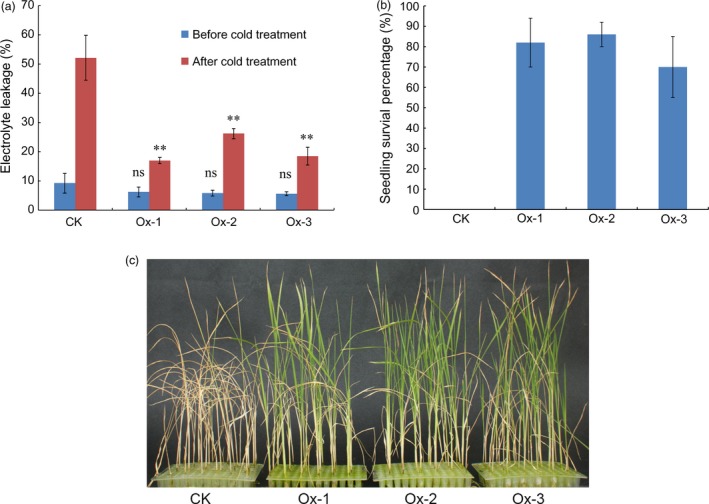
Performance of Os09g0410300 over‐expression transgenic lines and nontransgenic wild‐type plants under cold stress. (a) Electrolyte leakages before cold treatment and after cold treatment for 3 days; (b) seedling survival percentage on the seventh day during recovery at ambient conditions after cold treatment; (c) a picture showing the differences in cold tolerance on the seventh day during recovery at ambient conditions after cold treatment. CK represents nontransgenic wild‐type plants; Ox‐1, Ox‐2 and Ox‐3 represent the three independent over‐expression transgenic lines, respectively. ns: no significant difference in electrolyte leakage compared with that of CK (*P > *0.05) before cold treatment based on *t*‐test. **: Significant difference in electrolyte leakage compared with that of CK (*P *<* *0.01) after cold treatment based on *t*‐test.

To characterize seedling cold tolerance of transgenic lines, three‐leaf stage seedlings of the over‐expression transgenic plants and nontransgenic wild‐type plants were subjected to cold stress in a growth chamber. No significant difference in electrolyte leakage was observed between transgenic plants and nontransgenic wild‐type plants under normal condition (*P* > 0.05). However, electrolyte leakage in all transgenic lines was significantly lower than that in nontransgenic wild‐type plants after cold treatment at 5 °C for 3 days (*P *<* *0.01) (Figure 5a). Seedling survival percentages of the transgenic plants and the wild‐type plants were also measured after cold treatment. None of nontransgenic seedlings survived when they were transferred to ambient conditions for 7 days after cold treatment at 5 °C for 5 days, while the seedling survival percentages for the three transgenic lines were over 70% (Figure 5b and c). These results demonstrate that over‐expression of Os09g0410300 can enhance cold tolerance at the seedling stage in rice, suggesting that Os09g0410300 is the functional gene of *qCTS‐9*.

## Discussion

In the present study, we applied the strategy of combining QTL mapping and genomewide expression profiling to identify candidate genes associated with cold tolerance at the seedling stage within the interval of *qCTS‐9* in LTH. Using this approach, we were able to cut down the number of candidate genes from 58 predicted genes within *qCTS‐9* interval defined by QTL mapping to eight genes that showed differential expression in LTH under cold stress. Among the eight candidate genes, only the gene Os09g0410300 exhibited different expression patterns between LTH and SHZ‐2 after cold treatment (Table 2). Expression analysis of the gene in RI lines derived from LTH and SHZ‐2 exhibited significantly positive correlation between cold induction of Os09g0410300 and seedling cold tolerance (Figure 2). In addition, the sequence analysis revealed five SNPs and one InDel in the promoters of Os09g0410300 between LTH and SHZ‐2, which may result in their difference in response to cold stress. Genotyping RIL population by InDel marker based on the insertion–deletion polymorphism in the promoter region of Os09g0410300 also exhibited the significantly association between marker genotypes and cold tolerance at the seedling stage (Table S5). Further, Os09g0410300 over‐expressing plants showed lower electrolyte leakage and higher seedling survival percentage compared with the wild‐type plants under cold stress. Thus, based on these results, we conclude that Os09g0410300 is the functional gene of *qCTS‐9* contributing to the cold tolerance at the seedling stage in LTH.

According to the RAP database (http://rapdb.dna.affrc.go.jp) (Sakai *et al*., 2013), Os09g0410300 encodes a hypothetical protein with unknown function. Sequence alignment in NCBI database also indicated no significant known domains in it, but it was similar to a few proteins with unknown function. Therefore, the function of this gene is largely unknown. The only clue for its function comes from a previous study. This study reported that Os09g0410300 might interact with and be phosphorylated by Brassinosteroid Insensitive 1 (BRI1) in rice (Hirabayashi *et al*., 2004), a plasma membrane receptor kinase involved in brassinosteroid (BR) signalling pathway (Wang *et al*., 2001). Many studied have demonstrated that BRs play important roles in protecting plants from a variety of environmental stresses including cold stress (Bajguz and Hayat, 2009; Fariduddin *et al*., 2014). It was reported that application of 24‐epibrassinoslide (the active form of BR) could improve cold tolerance at the seedling stage in maize (Singh *et al*., 2012). In rice, BR promoted cell elongation in young seedlings, seed germination and the early growth of seedling at low temperature (15 °C) (Fujii and Saka, 2001). The knockout mutant of *OsGSK1*, a rice ortholog of BR signalling negative regulator, *Brassinosteroid Insensitive 2* (*BIN2*), showed enhanced tolerance to cold stress (Koh *et al*., 2007). Other studies also demonstrated the positive effects of BR on cold stress by modulation of the genes in BR synthesis or signal pathways (Divi and Krishna, 2010; Qu *et al*., 2011). Therefore, we deduce that Os09g0410300 may exert function on cold tolerance at the seedling stage in rice through its involvement in BR signalling pathway by interacting with BRI1. However, further study is needed to confirm this possibility. So far, most of the implications of BR in plant cold tolerance are based on physiological phenotypes, and the molecular mechanisms of BR in cold tolerance are limited. Studies on Arabidopsis implicated that BRs might enhance plant cold tolerance through the C‐repeat binding factors (CBF) pathway (Kurepin *et al*., 2013), but more detailed studies are needed. The functional confirmation of Os09g0410300 on cold tolerance at the seedling stage and its involvement in BR signalling pathway may help understand the mechanism of BRs on cold tolerance at the seedling stage in rice.

In conclusion, we employed an integrated approach of combining QTL mapping and genomewide differential expression profiling to identify Os09g0410300 as the candidate gene of *qCTS‐9* in the present study. Based on the significant correlations between cold induction of Os09g0410300, insertion–deletion polymorphism in the promoter of Os09g0410300 and seedling cold tolerance, and particularly, the enhanced cold tolerance in Os09g0410300 over‐expression transgenic plants, we can deduce that Os09g0410300 is the gene contributing to cold tolerance at the seedling stage underlying *qCTS‐9*. To our knowledge, Os09g0410300 is a novel gene responsible for cold tolerance at the seedling stage in rice. Our results suggest that integrating QTL mapping with genomewide differential expression profiling is an efficient approach to identify the genes associated with complex traits such as cold tolerance in rice. As *qCTS‐9* was detected in different germplasm and under different cold environments using different evaluation indicators (Liu *et al*., 2010; Zhang *et al*., 2014), the cloning and functional confirmation of Os09g0410300 will provide a promising target for cold‐tolerant rice breeding through transgenic method and MAS using molecular markers. Development of the gene‐specific marker ID410300 in the present study would increase the accuracy of MAS for cold tolerance at the seedling stage in rice. However, it is unknown how Os09g0410300 exerts its function on cold tolerance at the seedling stage. Based on the previous study (Hirabayashi *et al*., 2004), we expect that Os09g0410300 may be involved in BR pathway by interacting with BRI1, but further study is needed to confirm this deduction.

## Experimental procedures

### Plant materials

In the present study, LTH (a cold‐tolerant japonica variety), SHZ‐2 (a cold‐sensitive indica variety) and their derived RIL population consisting of 204 lines in F_8_ generation (Zhang *et al*., 2014) were used for mapping and gene expression analysis. The japonica variety ZhongHua‐11 (ZH‐11) was used for gene transformation.

### Cold treatment and evaluation of cold tolerance at the seedling stage

To conduct microarray and gene expression analysis, the germinated seeds of LTH and SHZ‐2 were sown in plastic trays (41 × 26.5 × 7.5 cm) filled with fine field soil. Materials were planted in 13 rows in a tray with 11 plants per row. The seedlings were allowed to grow for 14 days at 26 °C, and then, the three‐leaf stage seedlings were placed in a Conviron PGV36 growth chamber (Controlled Environments Ltd., Winnipeg) maintained at a constant temperature of 8 °C, with a 13‐h day length of 126 μmol/m^2^/s light intensity and a relative humidity of 80 ± 5%. Control seedlings were grown under the same conditions, except the temperature was 26 °C. The second and third leaves of seedlings were harvested at 6, 12, 24 and 48 h after cold treatment, quickly frozen in liquid nitrogen and stored at −80 °C until use. To investigate relationship between the expression of the candidate gene Os09g0410300 and the cold tolerance at the seedling stage under cold treatment, 20 RI lines and their parents LTH and SHZ‐2 were subjected to a constant temperature of 11 °C and the leaves of seedlings were harvested at 12 h after cold treatment for gene express analysis. The cold tolerance of the RI lines and their parents were evaluated after cold treatment for 5 days using the scale of 1 (tolerant, all leaves normal, no apparent visual injury) to 9 (sensitive, all leaves rolled and wilted, seedlings apparently dead) (Xu and Shen, 1989). Three biological replicates were performed for each pair of cold treatment and control at each time point.

To delimitate the confidence interval of *qCTS‐9* and validate the association between the difference in Os09g0410300 promoter sequence and the cold tolerance, the phenotypic data of cold tolerance in RIL population in our previous study (Zhang *et al*., 2014) were used for analysis.

To assess cold tolerance of the transgenic rice plants, the rice seedlings were cultivated in Kimura B solution. The three‐leaf stage seedlings of transgenic rice plants and nontransgenic wild‐type plants were subjected to 5 °C low temperature in a growth chamber, with a 13‐h day length of 126 μmol/m^2^/s light intensity and a relative humidity of 80 ± 5%. After cold treatment for 3 days, the second leaves were harvested to assay electrolyte leakage as described by Huang *et al*. (Huang *et al*., 2009). The electrolyte leakage of second leaves before cold treatment was taken as control. After cold treatment for 5 days, the temperature in the growth chamber was increased gradually to 25 °C within 24 h with a speed of 0.5 °C/h, and then, the seedlings were transferred to ambient conditions in the greenhouse for recovery. The survival percentage of seedlings was counted on the seventh day of recovery, and the cold tolerance at the seedling stage was expressed as seedling survival percentage.

### Delimitation of confidence interval of *qCTS‐9*

In our previous study, the cold‐tolerant QTL *qCTS‐9* was mapped using the RIL population consisting of 204 RI lines and 315 molecular markers (Zhang *et al*., 2014). To delimitate the confidence interval of *qCTS‐9*, more SSR markers located in the putative QTL region were used for genotyping of the RIL population. Composite interval mapping (CIM) was conducted to map *qCTS‐9* using Windows QTL Cartographer 2.5 (Wang *et al*., 2007). Based on the CIM result, the confidence interval of *qCTS‐9* was delimitated based on ‘1‐LOD support interval’ (Lander and Botstein, 1989).

### Genomewide differential expression profiling of LTH under cold stress

Agilent Rice Oligo Microarray 4 × 44 K (Agilent‐015058, Agilent Technologies, Santa Clara, CA, USA) was used for global gene expression analysis. Microarray assays and data analysis were conducted as described in our previous study (Zhao *et al*., 2015). DEGs were identified using the combined criteria of threefold change and a cut‐off of *q*‐values (≤0.05) in SAM based on three biological replicates.

The original microarray data set of this study has been uploaded to NCBI's Gene Expression Omnibus (http://www.ncbi.nih.gov/geo/) under GEO series number GSE58132.

### qRT‐PCR assays

qRT‐PCR was used to quantify the expression level of candidate genes in LTH, SHZ‐2 and RI lines under cold stress. The qRT‐PCR was conducted using Ex‐Taq SybrGreen PCR Mix (Takara, Japan) in Roche 480 real‐time PCR machine (Roche, Germany). The same RNA samples used in microarray assays were used in qRT‐PCR to confirm the microarray results and compare the expression patterns of the candidate genes in LTH and SHZ‐2. All samples were conducted in three biological replicates. The primers used for qRT‐PCR were listed in Table S4.

### Cloning of promoter and predicted full‐length cDNA of candidate gene

The promoter and predicted full‐length cDNA of candidate gene (Os09g0410300) was cloned by PCR with primers designed according to the annotation of RAP database (http://rapdb.dna.affrc.go.jp) (Sakai *et al*., 2013). The primers used for promoter amplification were PromoterF: 5′‐aggcaattcctcatgcagtagc‐3′, PromoterR: 5′‐tgcatcaagtcctttatggtgaa‐3′. The primers used to amplify full‐length cDNA were cDNAF: 5′‐aaaagtcgacgatgcagctcttttattattcttg‐3′, cDNAR: 5′‐aaaagaattcaaatcaagctagcatgaggcct‐3′. Primers cDNAF and cDNAR were also used to construct over‐expression vector of Os09g0410300. Promoters and cDNA sequences were amplified from LTH and SHZ‐2. The PCR products were cloned into pMD‐18T vector (Takara, Japan) and sequenced by Sangon Biotech (Shanghai, China). Cis‐element analysis was conducted in PLACE (http://www.dna.affrc.go.jp/PLACE/) (Higo *et al*., 1999).

### Generation of Os09g0410300 over‐expression transgenic rice

Predicted full length of Os09g0410300 was cloned into binary expression vector pHQSN and driven by cauliflower mosaic virus‐35S (CaMV‐35S) promoter and the castor bean catalase intron (35S/Intron) as described by Li (Li *et al*., 2011). Rice variety ZH‐11 was transformed by the Agrobacterium‐mediated method (Hiei *et al*., 1994). Twelve T_0_ transgenic lines were obtained. PCR and qRT‐PCR were conducted to validate the over‐expression of Os09g0410300 in both T_1_ and T_2_ transgenic rice plants. Three T_2_ homozygous transgenic lines showed obviously up‐regulation of Os09g0410300 were selected for further phenotyping.

## Conflict of interest

The authors declare that they have no competing interests.

## Supporting information


**Table S1** Annotated genes within the interval of *qCTS‐9*.

**Table S2** The expression change of Os09g0410300 and cold tolerance in the RI lines under the cold treatment.

**Table S3** Cis‐element difference in Os09g0410300 promoters between LTH and SHZ‐2.

**Table S4** List of primers used in the present study.

**Table S5** Associations between the InDel marker ID410300 and the cold tolerance at the seedling stage in RIL population under two distinct cold environments.
