# Supplementary material for: A novel functional gene associated with cold tolerance at the seedling stage in rice
Source: Plant Biotechnol J. 2017 Mar 30;15(9):1141–8. doi: 10.1111/pbi.12704 (PMC5552475; doi:10.1111/pbi.12704)
Supplement: Supplementary file 5 — Table S5 Associations between the InDel marker ID410300 and the cold tolerance at the seedling stage in RIL population under two distinct cold environments. [file PBI-15-1141-s001.docx]

Table S5 Associations between the InDel marker ID410300 and the cold tolerance at the seedling stage in RIL population under two distinct cold environments

| Marker ^a^ | CT-R ^b^ | CT-WP ^b^ |
| --- | --- | --- |
| ID410300 | 12.9**** | 7.9**** |

^a^ ID410300 is an InDel marker designed based on the 2 bp insertion-deletion polymorphism in the promoter of Os09g0410300 between LTH and SHZ-2.

^b^ The phenotypic data of cold tolerance is collected from our previous study (Zhang et al. 2014). CT-R: Evaluation of cold tolerance was conducted under 9℃ cold water irrigation at the International Rice Research Institute, the Philippines. Under cold water irrigation, only the root of seedling was exposed to the cold stress and the symptom observed on cold sensitive seedlings is leaf yellowing; CT-WP: Evaluation of cold tolerance was conducted in 11℃ low temperature growth chamber at Rice Research Institute of Guangdong Academy of Agricultural Sciences, China. In the low temperature growth chamber, whole plants suffered from cold stress and the cold sensitive seedlings develop leaf rolling or wilting symptoms.

The value is the percent phenotypic variation explained by ID410300 in RIL population. **** indicates the significance level at *p* < 0.0001.
